# Supplementary material for: A meta-analysis of epigenome-wide association studies in Alzheimer’s disease highlights novel differentially methylated loci across cortex
Source: Nat Commun. 2021 Jun 10;12:3517. doi: 10.1038/s41467-021-23243-4 (PMC8192929; doi:10.1038/s41467-021-23243-4)
Supplement: Supplementary file 4 — Reporting Summary [file 41467_2021_23243_MOESM4_ESM.pdf]

nature research

Corresponding author(s): Katie Lunnon

Last updated by author(s): 18th February 2021

Reporting Summary

Nature Research wishes to improve the reproducibility of the work that we publish. This form provides structure for consistency and transparency in reporting. For further information on Nature Research policies, see Authors & Referees and the Editorial Policy Checklist .

Statistics

For all statistical analyses, confirm that the following items are present in the figure legend, table legend, main text, or Methods section.

n/a	Confirmed	
☐	☒	The exact sample size (*n*) for each experimental group/condition, given as a discrete number and unit of measurement
☐	☒	A statement on whether measurements were taken from distinct samples or whether the same sample was measured repeatedly
☐	☒	The statistical test(s) used AND whether they are one- or two-sided<br>*Only common tests should be described solely by name; describe more complex techniques in the Methods section.*
☐	☒	A description of all covariates tested
☐	☒	A description of any assumptions or corrections, such as tests of normality and adjustment for multiple comparisons
☐	☒	A full description of the statistical parameters including central tendency (e.g. means) or other basic estimates (e.g. regression coefficient) AND variation (e.g. standard deviation) or associated estimates of uncertainty (e.g. confidence intervals)
☐	☒	For null hypothesis testing, the test statistic (e.g. *F*, *t*, *r*) with confidence intervals, effect sizes, degrees of freedom and *P* value noted<br>*Give P values as exact values whenever suitable.*
☒	☐	For Bayesian analysis, information on the choice of priors and Markov chain Monte Carlo settings
☐	☒	For hierarchical and complex designs, identification of the appropriate level for tests and full reporting of outcomes
☐	☒	Estimates of effect sizes (e.g. Cohen's *d*, Pearson's *r*), indicating how they were calculated

Our web collection on statistics for biologists contains articles on many of the points above.

Software and code

Policy information about availability of computer code

Data collection	N/A
Data analysis	Statistical analysis was performed in R 3.5.2 and Bioconductor 3.8, Python. R Packages used were wateRmelon 1.26.0, minfi 1.28.4, CETS 0.99.2, sva 3.30.1, Meta 4.10.0, nlme 3.1.142, bacon 1.10.1, glmnet 2.0-18. Python package comb-p 33.1.1. We have also utilized GREAT 4.0.4 and REViGO (Gene Ontology: Jan 2017 monthly release, UniProt-to-GO mapping file: 15 Mar 2017). We have developed an interactive tool, which can present summary statistics, which is available from our website: www.epigenomicslab.com/ad-meta-analysis/. All scripts for data analyses performed in this manuscript can be found at github.com and a DOI for this has been generated in Zenodo and added.

For manuscripts utilizing custom algorithms or software that are central to the research but not yet described in published literature, software must be made available to editors/reviewers. We strongly encourage code deposition in a community repository (e.g. GitHub). See the Nature Research guidelines for submitting code & software for further information.

Data

Policy information about availability of data

All manuscripts must include a data availability statement. This statement should provide the following information, where applicable:

- Accession codes, unique identifiers, or web links for publicly available datasets
- A list of figures that have associated raw data
- A description of any restrictions on data availability

The data supporting the findings of this study are available within the article, Supplementary Information or from the authors upon request. Some of the datasets are also available on GEO including London 1 data (GSE59685), London 2 data (GSE105109), Mount Sinai data (GSE80970), Arizona 1 TG data (GSE134379), Arizona 2 TG data (GSE109627) and Munich data (GSE66351). Hyperlinks to all the datasets are provided in the Data Availability Statement.

Field-specific reporting

Please select the one below that is the best fit for your research. If you are not sure, read the appropriate sections before making your selection.

☒ Life sciences ☐ Behavioural & social sciences ☐ Ecological, evolutionary & environmental sciences

For a reference copy of the document with all sections, see nature.com/documents/nr-reporting-summary-flat.pdf

Life sciences study design

All studies must disclose on these points even when the disclosure is negative.

Sample size	Sample sizes were provided with data from GEO and data providers. Final numbers were determined after sample quality control. All relevant available EWAS datasets on GEO were utilized for the discovery meta-analysis if they had (a) Braak stage available, (b) >50 unique samples analyzed and (c) were analyzed using the Illumina Infinium 450K array. This provided data from >1,408 unique individuals, representing the largest meta-analysis of Braak stage to date.
Data exclusions	Samples were excluded from further steps if (a) the mean background intensity of negative probes < 1,000, (b) the mean detection P values > 0.005, (c) the mean intensity of methylated or unmethylated signals were three standard deviations above or below the mean, (d) the bisulfite conversion efficiency < 80%, (e) there was a mismatch between reported and predicted sex, or (f) the 65 single nucleotide polymorphism (SNP) probes on the array show a modest level of correlation (0.65) between two samples (whereby the sample with the higher Braak score was retained). Sample exclusion was performed using the pfilter function within the wateRmelon package, samples with a detection P > 0.05 in more than 5% of probes were excluded. These exclusion criteria were pre-established.
Replication	Replication was performed using two cohorts. (1) The "Munich" cohort" from Neurobiobank Munich (NBM), which had bulk PFC 450K array data from 45 donors, and 450K array data from fluorescence-activated cell sorting (FACS) isolated neuronal and non-neuronal (glial) populations from the occipital cortex (OC) from 26 donors. (2) The "BDR cohort", which had bulk PFC 450K array data from 590 donors.
Randomization	Samples were randomized as part of the original studies which went into the meta-analysis.
Blinding	All data for the study had already been collected. During the data quality control (QC) and pre-processing stages of the analysis the investigators were blinded to the outcome variable (Braak stage)

Reporting for specific materials, systems and methods

We require information from authors about some types of materials, experimental systems and methods used in many studies. Here, indicate whether each material, system or method listed is relevant to your study. If you are not sure if a list item applies to your research, read the appropriate section before selecting a response.

Materials & experimental systems

n/a	Involved in the study	
☒	☐	Antibodies
☒	☐	Eukaryotic cell lines
☒	☐	Palaeontology
☒	☐	Animals and other organisms
☐	☒	Human research participants
☒	☐	Clinical data

Methods

n/a	Involved in the study	
☒	☐	ChIP-seq
☒	☐	Flow cytometry
☒	☐	MRI-based neuroimaging

Human research participants

Policy information about studies involving human research participants

Population characteristics	Braak 0-II - 189 male/163 female - Age (SD) 81.47 (7.98)<br>Braak III-IV - 238 male/401 female - Age (SD) 87.07 (4.99)<br>Braak V-VI - 200 male/333 female - Age (SD) 84.99 (7.5)
Recruitment	Participants were recruited to relevant brain banks and selected for cohort analyses by original authors.
Ethics oversight	Ethical approval for the study was granted from the University of Exeter Medical School Research Ethics Committee (approval number 13/02/009).

Note that full information on the approval of the study protocol must also be provided in the manuscript.
